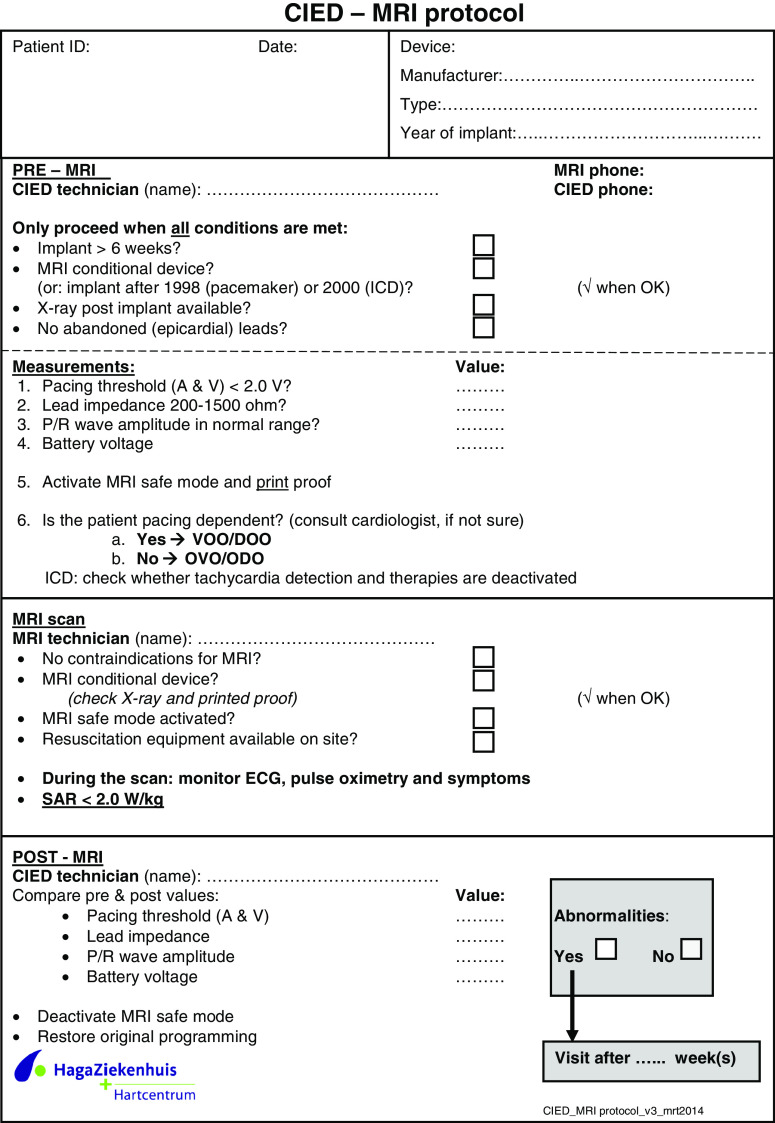# MRI and cardiac implantable electronic devices; current status and required safety conditions

**DOI:** 10.1007/s12471-014-0544-x

**Published:** 2014-04-15

**Authors:** A. W. M. van der Graaf, P. Bhagirath, M. J. W. Götte

**Affiliations:** Department of Cardiology, Haga Teaching Hospital, Leyweg 275, 2545 CH The Hague, the Netherlands

**Keywords:** MRI, Cardiac implantable electronic devices, Pacing

## Abstract

Magnetic resonance imaging (MRI) has evolved into an essential diagnostic modality for the evaluation of all patient categories. This gain in popularity coincided with an increase in the number of implanted cardiac implantable electronic devices (CIEDs). Therefore, questions arose with regard to the MRI compatibility of these devices. Various investigators have reported the harmless performance of MRI in patients with conventional (non-MRI conditional) devices. The recently published European Society of Cardiology (ESC) guidelines on cardiac pacing and cardiac resynchronisation therapy (CRT) indicate that MRI can be safely performed in patients with an implanted pacemaker or ICD (MRI conditional or not), as long as strict safety conditions are met. This is a major modification of the former general opinion that patients with a pacemaker or ICD were not eligible to undergo MRI. This review paper attempts to elucidate the current situation for practising cardiologists by providing a clear overview of the potential life-threatening interactions and discuss safety measures to be taken prior to and during scanning. An overview of all available MRI conditional devices and their individual restrictions is given. In addition, an up-to-date safety protocol is provided that can be used to ensure patient safety before, during and after the scan.

*Key points*

• *Historically, MRI examination of patients with a CIED has been considered hazardous.*

• *Ongoing advances in technology and increasing usage of MRI in clinical practice have led to the introduction of MRI conditional CIEDs and to more lenient regulations on the examination of patients with non-conditional CIEDs.*

• *MRI investigations can be performed safely in selected patients when adhering to a standardised up-to-date safety protocol.*

## Introduction

Historically, magnetic resonance imaging (MRI) examination of patients with a cardiac implantable electronic device (CIED) has been considered hazardous due to potential life-threatening interaction between the MRI scanner and the pacemaker or internal cardioverter defibrillator (ICD) [1, 2]. To increase patient safety and to anticipate the growing clinical need for MRI, an increasing number of MRI conditional CIEDs have become available [3].

However, the recently published European Society of Cardiology (ESC) guidelines on cardiac pacing and cardiac resynchronisation therapy (CRT) [4] state that MRI can be safely performed in patients with an implanted pacemaker or ICD, irrespective of the MRI conditional or non-specific MRI conditional design, as long as strict safety conditions are met.

In order to clarify this somewhat confusing situation, this review paper provides an overview of the currently available data related to CIEDs and MRI, and attempts to offer an up-to-date and clinically useful summary for the practising cardiologist. In addition, a safety protocol applicable for patients with a CIED is provided that can be used to ensure a patient’s safety before, during and after an MRI scan.

## MRI system

### Technical background

The fundamental components of an MRI system are the main magnet coils, three gradient coils and an integral radiofrequency transmitter coil. The main magnet coils generate a strong, constant magnetic field. The strength of this magnetic field is expressed in Tesla.

Mounted inside the main magnet, three gradient coils generate gradient magnetic fields that are rapidly switched on and off. The integral radiofrequency transmitter coil produces a radiofrequency magnetic field, used to deliver energy to a population of protons. The static magnetic field and radiofrequency field combine to generate magnetic resonance signals that are spatially localised and encoded by the gradient magnetic fields [5].

Receiving coils capture the energy released by resonating hydrogen protons. Subsequent analysis results in high-grade tissue characterisation and reconstruction of a detailed image.

The amount of energy administered to an individual is expressed in SAR (specific absorption rate). SAR values vary from 2.0 to 4.0 W per kilogram (W/kg). For reference purposes, SAR values of a modern mobile telephone are approximately 0.9 W/kg [6].

Over time, the strength of the static magnetic field of MRI systems has increased from less than 0.5 T to clinical scanners operating at 7.0 T and even 9.4 T. Today, the vast majority of clinical MRI exams are performed using a 1.5 T system, while a rapid growth of 3.0 T systems is observed [7]. Since the SAR is proportional to the square of the static magnetic field strength, the amount of energy absorbed by a patient increases rapidly in a scanner operating at a higher field strength. Therefore, application of higher field strengths poses constraints on the total scan time or the imaging sequences used.

## Interaction between the MRI system and CIEDs

Pavlicek et al. [8] were the first (1983) to discuss the potential interaction between pacing devices and the NMR (at that time nuclear magnetic resonance) environment using ex-vivo pacemakers of deceased patients. These interactions can be subdivided into three categories.

### Mechanical forces

The constant static magnetic field strongly attracts different types of metal. Implantable devices usually contain a small amount of one or more of these metals and are considered to be ferromagnetic. Therefore, there is the possibility of movement of the implanted pacemaker.

A 6-week interval between implantation and MRI examination is advised in international guidelines in order to ensure sufficient encapsulation of the device [9].

### Induction (antenna function)

The several electromagnetic components used in MRI may cause electrical or thermal induction in implanted leads. Inducted electrical currents could initiate arrhythmias or lead to oversensing or undersensing of the pacemaker or ICD with potentially fatal consequences. In addition, since tissue near the lead tip has limited conductivity, energy will be converted to heat at this location. Subsequent thermal damage around the lead tip includes oedema or formation of scar tissue. In both situations, an increase in pacing thresholds or even complete loss of capture may occur [10].

### Defibrillators

The magnetic fields inside the MRI may have an unpredictable intermittent effect on the activity of the reed switch in a pacemaker or ICD. This may lead to either asynchronous pacing (reed switch closed) or unwanted inhibition of pacing in the presence of an open reed switch. In addition, the rapidly changing magnetic gradients can be registered as a life-threatening arrhythmia. The ICD can subsequently react with release of anti-tachycardia pacing (ATP) or shock [11]. In the presence of the main static magnetic field, the core of the transformer, necessary to charge the high-energy storage capacitor, tends to saturate. Thereby, the storage capacitor is prevented from charging. Although this reduces the risk for the deliverance of inappropriate shocks during the scan, the battery will lose life.

### Software interaction

Exposure to the electromagnetic fields may also directly affect or modify the electronic circuits and functional settings of the CIED. Use of external programmers may become impossible due to damaged electrical circuits inside the CIED.

### Implantable loop recorders (ILR)

Several studies have indicated that MRI scanning of ILR patients can be performed without harm to patient or device. However, signal artefacts that can be mistaken for a tachyarrhythmia are seen frequently [12, 13]. Of course, image artefacts arising from the presence of an IPL can degrade the image quality.

## Review of literature

### Non-conditional pacemakers and ICDs

In 1984, Fetter et al. [14] investigated the potential interactions using four pacemakers in vivo. The asynchronous (VOO) pacing mode was activated during the scan in one patient. The authors concluded that patients with single-chamber implantable pacemakers may undergo scanning with MRI, provided the patient is monitored during scanning and the risks of asynchronous pacing are taken into account.

Gimbel et al. (1996) performed an MRI scan with a field strength of 0.5 T in five patients [15]. A 2-s pause was observed in one pacemaker-dependent patient. The other patients were asymptomatic and did not report any discomfort. Fontaine et al. (1998) demonstrated the development of an irregular ventricular rhythm in a 69-year-old patient during RF pulsing on a 1.5 T scanner [16]. This rhythm terminated with the cessation of RF pulsing. The patient remained asymptomatic during the procedure.

In 2000, Sommer and co-workers examined 44 non-pacemaker-dependent patients 51 times in a 0.5 T MRI without any impairment of pacing function [17]. Vahlhaus (2001) and co-workers postulate in a paper that ‘the general policy of never exposing a patient with a pacemaker to MRI should be revised’ [18].

Bartsch et al. (2003) reported four MRI-associated deaths in paced patients undergoing MRI (1.5 T) assessment [19]. Importantly, none of these patients were pacemaker dependent and in some cases ventricular fibrillation was proven to be the cause of death.

In 2005, Irnich et al. questioned 30 legal medicine departments in Germany with respect to casualties with a fatal outcome of pacemaker patients during an MRI examination (0.5–1.5 T) between 1992 and 2001 [20]. Six fatal cases occurred, in three cases ventricular fibrillation (VF) was proven to be the cause of death. In the other three cases the pacemakers were removed from the deceased patient’s body and introduced in the MRI scanner. The pacemaker showed a magnetic asynchronous rate of 100/min. It was suspected that the asynchronous pacing had induced VF in these patients.

In the following years, several investigators collected and published data on MRI examinations in patients with conventional (non-MRI conditional) pacemakers and ICDs. Naehle et al. (2009) reported the safe scanning of 18 ICD patients at 1.5 T [21]. No significant change in pacing capture threshold, lead impedance or serum troponin I was observed. In two MRI examinations, oversensing of radiofrequency noise as ventricular fibrillation occurred. However, no attempt at therapy delivery was made.

More recently, Nazarian et al. (2011) published the most elaborate study [22]. A total of 555 MRI scans (1.5 T) were performed in 438 patients with a CIED (54 % pacemakers, 46 % defibrillators). Of the 555 MRI examinations, 222 (40 %) were of the brain, 122 (22 %) were of the spine, 89 (16 %) were of the heart, 72 (13 %) were of the abdomen or pelvis, and 50 (9 %) were of an extremity. Only small changes in programming, sensing and impedance were reported. The observed changes did not ever require device revision. Unfortunately, no data are available on the possible effects of repeated MRI examinations in individual patients. However, it must be noted that although a large number of different models were studied, the numbers for each individual model were small. Patients were enrolled during a long period of time (from 2003 to 2010) and CIEDs were constantly evolving. Last but not least, a control group was lacking in this study.

Despite these limitations, it is only this paper that is exclusively referred to in the recently published ESC guidelines on cardiac pacing and CRT. MRI conditional CIEDs were developed in order to decrease potential life-threatening hardware interactions.

### MRI conditional pacemakers and ICDs

These systems contain specially developed components, tested and approved for usage in an MRI environment. Improved lead design reduces the risk of complications, such as lead-tip heating. In addition to the modified hardware design, MRI conditional pacemaker systems are provided with a special MRI software mode. Upon activation, the most appropriate settings are switched on automatically. These settings include bipolar stimulation instead of unipolar pacing and an increased electrical output. Specific filters inside the device prevent sensing of external non-cardiac signals. Furthermore, recording of arrhythmic episodes is temporarily disabled during the scan.

It must be noted that due to constant technological improvements the size of the available implantable pulse generators has substantially decreased and leads have become more sophisticated. Together with a reduction of ferromagnetic components in contemporary CIEDs, the chance of hardware interactions is therefore substantially diminished.

Medtronic commercialised the first MRI conditional pacemaker system (Enrhythm MRI®) in 2008. Several trials reported the safe performance of MRI scans in patients with an implanted Enrhythm MRI system [23, 24]. However, scanning of the thorax region was prohibited. Different manufacturers initiated comparable clinical trials to demonstrate the safety of their own MRI conditional devices. Currently, several MRI conditional pacemakers are commercially available that enable full body MRI examination. Table 1 provides a complete overview of currently available MRI conditional pacemakers and ICDs.

**Table 1:** Currently available MRI conditional pacemakers and ICDs

	Medtronic		St Jude Medical	Biotronik		Boston
	Pacemaker		Pacemaker	Pacemaker	ICD	Pacemaker
	Enrhythm MRI / Revo MRI	Advisa MRI	Accent MRI	Evia ProMRI	Lumax ProMRI / Iforia ProMRI / Ilesto ProMRI	Ingenio MRI
Restrictions	Full body	Full body	Full body	No chest scans	No chest scans	Full body
MRI machine	Cylindrical bore magnet, clinical MRI systems with a static magnetic field of 1.5 T					
SAR limitation (whole body)	≤2.0 W/kg	≤2.0 W/kg	≤4.0 W/kg	≤2.0 W/Kg	≤2.0 W/Kg	≤2.0 W/Kg
Maximum number of scans	No	No	No	Yes: each scan ≤30 min	Yes: each scan ≤30 min	No
				Total scan time max: 10 hours	Total scan time max: 10 hours	
Published clinical trial evidence on MRI environment	Yes	Yes	No	No	No	No

### Current clinical trials

**Fig. 1:**
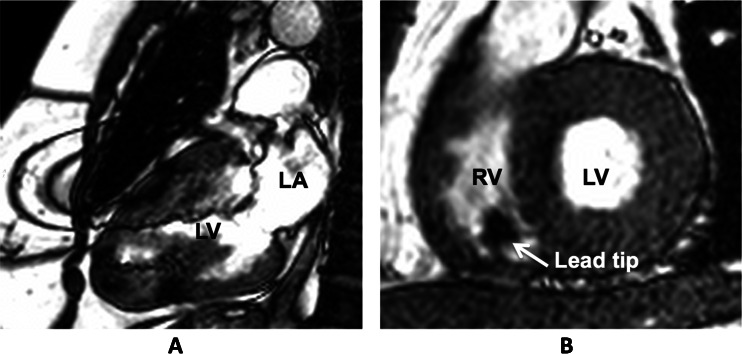
Steady state free precession CINE images. Typical artefacts as can be observed in patients with an implanted impulse generator (**a**) and pacing leads (**b**). Note that the image quality in the short axis cine (**b**) is sufficient to allow reliable calculation of left ventricular ejection fraction

An overview of clinical trials currently enrolling patients is given in Table 2. Apart from demonstrating the safety of a device in an MRI environment, contemporary studies are more focused on the influence on image quality and artefacts caused by the CIED (Fig. 1).

**Table 2:** Current clinical trials on MRI conditional pacing devices

	Medtronic	St Jude Medical	Biotronik	Boston
Study	Advisa II	Accent	ProMRI AFFIRM	Samurai
Number of participating sites	40	80	21	45
Estimated number of patients	270	800	245	363
Implantable pulse generator–lead	Advisa–5076	Accent–Tendril	Evia/Entovis–Safio S	Image Ready System
Estimated date of completion	October 2014	July 2014	December 2013	July 2014

### Biventricular (CRT) devices

The presence of three leads in patients treated with biventricular (CRT) therapy increases the risk of interactions between MRI and the pacing system. Only a small and inconsistent amount of literature on MRI examinations in CRT patients is available at this time. The scanning of patients with an implanted biventricular pacing device is strongly discouraged.

## Risk assessment

### Pacemaker dependent patients

Patients with an implanted pacemaker or ICD and an absent or insufficient intrinsic heart rhythm constitute a special group, because potential interaction between pacemaker and MRI can have life-threatening consequences. It must be ascertained that the potential benefit of an MRI examination outweighs the potential risks and no diagnostic alternative is available. Close monitoring of the patient using pulse oximetry is warranted.

### MRI conditional markers and MRI mode

**Fig. 2:**
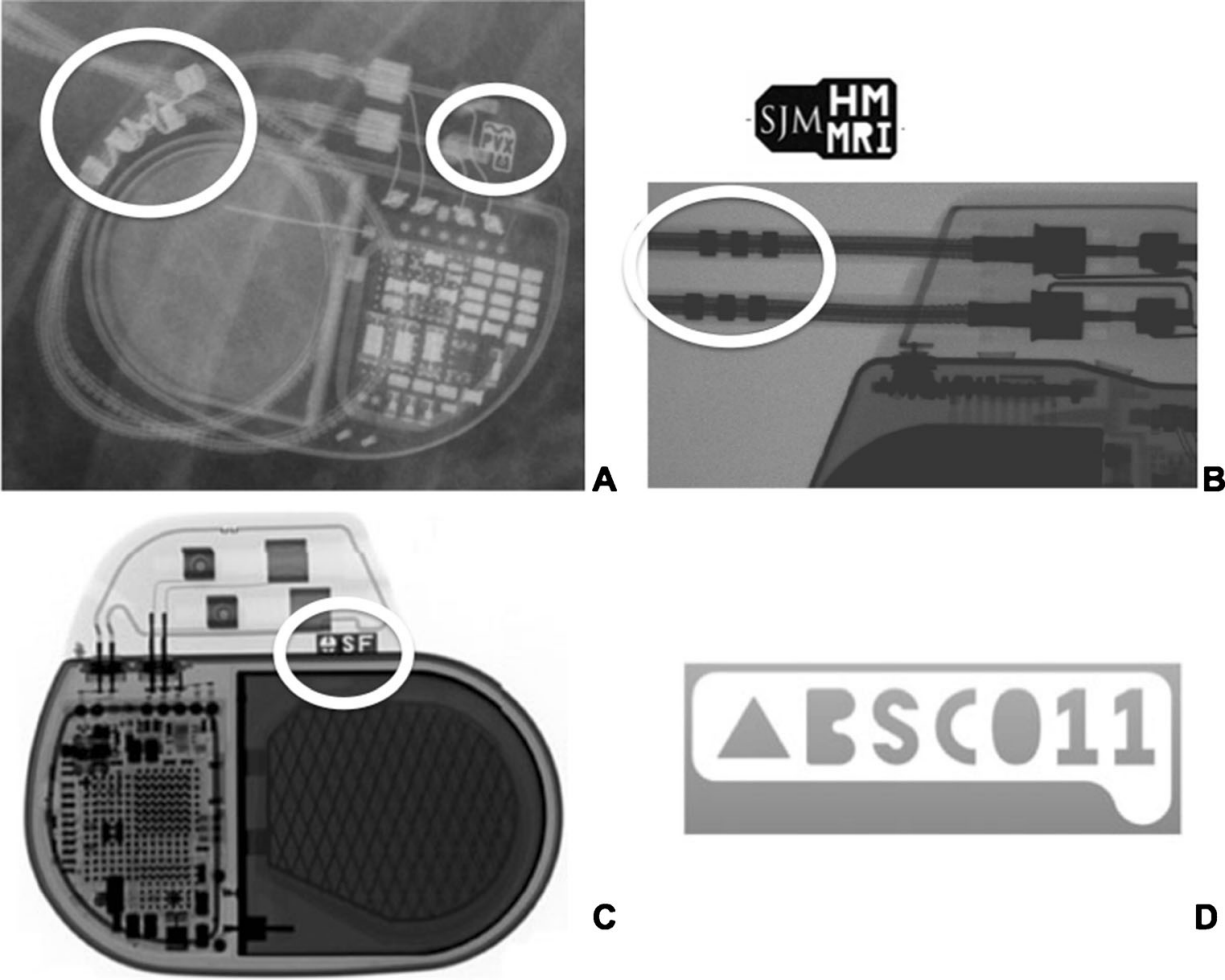
Vendor-specific markers on MRI conditional pacing systems as can be identified on routine chest X-rays. **a** Medtronic, **b** St. Jude Medical, **c** Biotronik, **d** Boston Scientific

Vendor-specific markers on CIEDs can facilitate the identification of MRI conditional systems on routine chest X-rays (Fig. 2). Certain CIEDs contain a selectable MRI mode, which automatically activates the most appropriate settings. After the scan, the original settings can be reprogrammed easily. Other (non-conditional) CIEDs mandate a manual selection of some of these settings and reprogramming.

### Various combinations of implantable pulse generators and leads

A more complex situation occurs when implantable pulse generators and leads from different vendors, each bearing their own MRI restrictions, are combined together in individual patients. In this case the MRI compatibility of all parts of the CIED needs to be ascertained. An individualised approach is mandatory in these patients. When abandoned pacing leads are present, for example after recent coronary bypass grafting (CABG), scanning is strongly discouraged. The absence of an implantable pulse generator increases the chance of current induction in the leads of these patients.

## Preparation and monitoring during MRI examination

An MRI examination of a patient with a CIED is preferably performed exclusively in centres with extensive experience and expertise in this area. Written informed consent should be obtained from the patient after extensive notification of the procedural risks. Application of a safety protocol (Appendix 1) and appropriate monitoring are mandatory to perform an MRI scan in patients with a CIED.

Prior to the examination, proper functioning of the CIED needs to be assured. The pacing threshold is one of the most important issues and should always be determined. An already elevated stimulation threshold (>2.0 V) increases the risk of loss of capture during the examination. Pacemaker output should be increased and the examination should be suspended in case of a severely elevated threshold and/or deviated lead impedance <200 ohms or >1,500 ohms.

During the examination careful monitoring of the patient using a heart rate monitor and pulse oximetry is warranted. The recorded ECG signal in the scanner is subject to disturbances and therefore unreliable. Pulse oximetry is not affected by the MRI scanner and should always be used to monitor the patient. Cardiopulmonary resuscitation equipment including a cardiac defibrillator, along with staff experienced in resuscitation, should be available on site.

The nature of the cardiac MRI examination, especially in pacemaker patients, mandates routine verbal communication between operator and patient during the scan and is of vital importance. During and after the MRI examination, the patient should be asked about any discomfort or complaints. When the MRI examination is finished, the original settings of the CIED should be restored after confirming that these are still safe and provide adequate margins. In order to exclude any late side effects or symptoms, a control visit (3–6 months after the scan) to the outpatient department may be recommended [25].

## Discussion

The recently published ESC guidelines on cardiac pacing and CRT somewhat trivialised the absolute necessity of MRI conditional CIEDs by stating that MRI can be safely performed in patients with an implanted pacemaker or ICD (MRI conditional or not), as long as strict safety conditions are met.

Despite abundant literature [26–31] reporting the harmless performance of MRI investigations in patients with conventional (non-MRI conditional) pacemakers and ICDs, it is still considered potentially hazardous. Only a limited number of prospective studies are available. Long-term studies are confounded by the use of several generations of CIEDs. The variety of tested devices in these studies affects the conclusions and decreases the clinical value.

Therefore, it remains difficult to state whether a specific conventional CIED can be introduced into the MRI room without possible consequences. For MRI conditional CIEDs, the possibilities, limitations and required safety measures to be taken are more uniform and described in detail.

In daily practice, an increasing number of clinicians are confronted with questions on MRI compatibility of CIEDs. The value of a safety protocol, approved by the cardiology and radiology department, cannot be stressed enough.

It is expected that the role of MRI in clinical decision-making will gain even more clinical importance. There is an emerging role for MRI in identifying arrhythmogenic substrates and this modality has an expanding role in guiding electrophysiological therapies.

Now that the patient’s safety seems to be conditionally guaranteed, a new challenge is the reduction of the impact of CIEDs on the image quality, especially with regard to the identification and modification of an arrhythmogenic substrate.

Finally, it is important to realise that almost all published data are only valid for scanning on 1.5 Tesla machines. The application of 3.0 Tesla machines is rapidly emerging, especially for orthopaedic and neurological patients, due to the higher signal-to-noise ratio (SNR). With the advance of 3.0 T machines, new technical challenges arise.

## Conclusion

MRI conditional CIEDs are designed to cope with the challenges introduced by the electromagnetic MRI environment. Various papers have demonstrated the safe and harmless performance of MRI examination in patients with CIEDs, MRI conditional or not. With appropriate monitoring and application of a safety protocol, MRI can be safely performed in patients with CIEDs. For patients equipped with a conventional CIED or those who are pacing dependent it must be ascertained that the potential benefits of an MRI examination outweigh the potential risks.